# The Synthesis and Characterization of Aromatic Hybrid Anderson–Evans POMs and their Serum Albumin Interactions: The Shift from Polar to Hydrophobic Interactions

**DOI:** 10.1002/chem.201502458

**Published:** 2015-11-03

**Authors:** Emir Al-Sayed, Amir Blazevic, Alexander Roller, Annette Rompel

**Affiliations:** a Institut für Biophysikalische Chemie Fakultät für Chemie, Universität Wien, Althanstraße 14, 1090 Wien (Austria) http://www.bpc.univie.ac.at; b Institut für Anorganische Chemie, Fakultät für Chemie Universität Wien, Währinger Straße 42, 1090 Wien (Austria)

**Keywords:** characterization, hydrophobic effect, polyoxometalates, synthesis, X-ray crystallography

## Abstract

Four aromatic hybrid Anderson polyoxomolybdates with Fe^3+^ or Mn^3+^ as the central heteroatom have been synthesized by using a pre-functionalization protocol and characterized by using single-crystal X-ray diffraction, FTIR, ESI-MS, ^1^H NMR spectroscopy, and elemental analysis. Structural analysis revealed the formation of (TBA)_3_[FeMo_6_O_18_{(OCH_2_)_3_CNHCOC_6_H_5_}_2_]**⋅**3.5 ACN (**TBA-FeMo_6_-bzn**; TBA=tetrabutylammonium, ACN=acetonitrile, bzn=TRIS-benzoic acid alkanolamide, TRIS–R=(HOCH_2_)_3_C–R)), (TBA)_3_[FeMo_6_O_18_{(OCH_2_)_3_CNHCOC_8_H_7_}_2_]**⋅**2.5 ACN (**TBA-FeMo_6_-cin**; cin=TRIS-cinnamic acid alkanolamide), (TBA)_3_[MnMo_6_O_18_{(OCH_2_)_3_CNHCOC_6_H_5_}_2_]**⋅**3.5 ACN (**TBA-MnMo_6_-bzn**), and (TBA)_3_[MnMo_6_O_18_{(OCH_2_)_3_CNHCOC_8_H_7_}_2_]**⋅**2.5 ACN (**TBA-MnMo_6_-cin**). To make these four compounds applicable in biological systems, an ion exchange was performed that gave the water-soluble (up to 80 mm) sodium salts Na_3_[FeMo_6_O_18_{(OCH_2_)_3_CNHCOC_6_H_5_}_2_] (**Na-FeMo_6_-bzn**), Na_3_[FeMo_6_O_18_{(OCH_2_)_3_CNHCOC_8_H_7_}_2_] (**Na-FeMo_6_-cin**), Na_3_[MnMo_6_O_18_{(OCH_2_)_3_CNHCOC_6_H_5_}_2_] (**Na-MnMo_6_-bzn**), and Na_3_[MnMo_6_O_18_{(OCH_2_)_3_CNHCOC_8_H_7_}_2_] (**Na-MnMo_6_-cin**). The hydrolytic stability of the sodium salts was examined by applying ESI-MS in the pH range of 4 to 9. Sodium dodecylsulfate–polyacrylamide gel electrophoresis (SDS-PAGE) showed that human and bovine serum albumin (HSA and BSA) remain intact in solutions that contain up to 100 equivalents of the sodium salts over more than 4 d at 20 °C. Tryptophan (Trp) fluorescence quenching was applied to study the interactions between the sodium salts and HSA and BSA at pH 5.5 and 7.4. The quenching constants were extracted by using Stern–Volmer analysis, which suggested the formation of a 1:1 POM–protein complex in all samples. It is suggested that the aromatic hybrid POM approaches subdomain IIA of HSA and exhibits hydrophobic interactions with its hydrophobic tails, whereas the Anderson core is stabilized through electrostatic interactions with polar amino acid side chains from, for example, subdomain IB.

## Introduction

Polyoxometalates (POMs) are polyanions made up of early transition metals in their highest oxidation states (d^1^ and d^0^) that are bridged by oxygen atoms.^1^ Changing their size, shape, or composition enables the tuning of POMs for different kinds of applications (e.g., catalysis,^2^–^4^ material science^5^–^7^ and medicine,^8^–^11^ bio- and nanotechnology,^12^–^15^ and macromolecular crystallography).^16^–^23^ The Anderson polyoxoanion is composed of six edge-sharing MO_6_ (M=W or Mo) octahedra that surround a central, edge-sharing heteroatom octahedron (XO_6_). The general structure can be subdivided into two categories: the nonprotonated A-type with central heteroatoms with high oxidation states and the general formula [X^*n*+^M_6_O_24_]^(12−*n*)−^ (M=Mo^6+^, W^6+^; X=heteroatom, e.g., Te^6+^, I^7+^) and the protonated B-type with the general formula [X^*n*+^M_6_O_18_]^(6−*n*)−^ (M=Mo^6+^, W^6+^; X=heteroatom, e.g., Ga^3+^, Cr^3+^, Fe^3+^) with heteroatoms in low oxidation states.^1^

Hybrid organic–inorganic POMs have been known for a long time, and make it possible to combine the inorganic POM with specific organic functionalities to give new materials with interesting properties.^24^–^26^ Hybrids based on the Anderson structure can be synthesized by attaching one or two tris(hydroxymethyl)methane derivatives (TRIS–R; (HOCH_2_)_3_C–R) to the planar metal oxide framework. The first report by Hasenknopf et al.^27^ described the grafting of R–C(CH_2_OH)_3_ (R=CH_3_, NO_2_, CH_2_OH) onto polyoxomolybdates with Ni^2+^, Zn^2+^, Fe^3+^, and Mn^3+^ as central heteroatoms. Since then, numerous reports on the further derivatization of the functional groups on the tripodal alcohols through imine and peptide bond formation have been reported, to give POMs with various properties.^28^–^32^ The classical method for obtaining a symmetric Anderson hybrid POM is a one-pot reaction in a polar organic solvent in the presence of octamolybdate, a salt of the central heteroatom and the organic ligand.^27^ The TRIS ligands were pre-functionalized before being introduced into the reaction mixture, that is, the organic ligand is formed first then incorporated during the constitution of the hybrid cluster. Further success of functionalizing the organic ligands in Anderson polyoxometalates by pre- or post-functionalization has recently been reviewed.^33^

The versatile applications of POMs in macromolecular X-ray crystallography has been reviewed.^19^ Several aspects make POMs ideal candidates for use as crystallization additives,^20^–^23^ especially the Anderson POM.^19^ It can be used as a phasing tool and only a few binding sites are necessary, whereas mononuclear heavy atoms must bind to multiple sites to provide useful phases, especially for large proteins.^34^–^36^ Furthermore, most POMs have high negative charges that make it possible to crosslink positive regions of several monomers through electrostatic interactions. This leads to the formation of new contacts and increases the chance of a long-range-order formation.^18^ In one instance, protein crystals have only been obtained in the presence of POMs, in the case of mushroom tyrosinase (*ab*PPO4, *ab*=*Agaricus bisporus*, PPO=polyphenol oxidase) for which protein crystals were only obtained in the presence of the [TeW_6_O_24_]^6−^ anion.^20^, ^21^ The interaction between POMs and proteins seems to be predominantly electrostatic, but hydrogen bonds, covalent bonds, π–π, and van der Waals interactions have also been observed. Encouraged by the successful use of the Anderson POM in protein crystallography, we here functionalized the archetype with aromatic ligands. Tryptophan fluorescence quenching was applied to investigate possible interactions between the aromatic ring grafted onto the Anderson POMs and the aromatic amino acids on the protein surface, with the aim of enabling different POM–protein interactions and thus using the aromatically TRIS-functionalized hybrid Anderson–Evans POMs reported herein as future additives in macromolecular crystallography.

## Results and Discussion

### Synthesis

**scheme 1:**
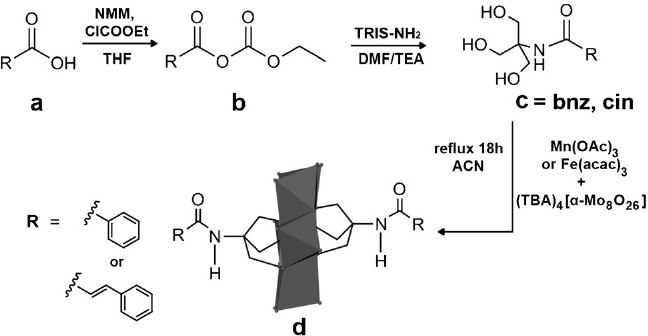
a)–c) Schematic illustration of the preparation steps of the organic ligands and d) the grafting onto the Anderson POM to give hybrid POMs with aromatic ligands. Legend: MoO_6_: grey octahedra; O: grey spheres. Hydrogen atoms are omitted for clarity.

The pre-functionalization of the organic ligands was achieved by using an established procedure.^37^ The organic acid (benzoic acid or *trans*-cinnamic acid) **a** (Scheme 1) was added to ethyl chloroformate in the presence of *N*-methylmorpholin (NMM) in THF to generate mixed anhydride **b**, which subsequently reacted with TRIS-NH_2_ in dimethylformamide (DMF) and triethylamine (TEA) to form alkanolamides (bzn and cin) **c** (the ^1^H NMR spectroscopic characterization of bzn and cin is given in Figures S3 and S4 in the Supporting Information). The bzn and cin ligands differ in their carbon chain length, which leads to more flexibility in the POMs that contain cin ligands upon interaction with proteins.

Fe^3+^ and Mn^3+^ were chosen as templating heteroatoms because both provide POM hybrids in good yields (86 % based on Mo).^38^ Compounds **TBA-FeMo_6_-bzn**, **TBA-FeMo_6_-cin**, **TBA-MnMo_6_-bzn**, and **TBA-MnMo_6_-cin** were obtained by heating Fe(acac)_3_ or Mn(OAc)_3_, (TBA)_4_[α-Mo_8_O_26_], and bzn or cin at reflux in acetonitrile for 18 h (Scheme 1d). All four compounds were isolated as TBA salts with poor water solubility (<3 mm). The solubility was increased by cation exchange with Na^+^ by mixing the TBA salts with NaClO_4_ for 30 min in ACN to form a precipitate of the Na salt.^28^ This led to the isolation of Na_3_[FeMo_6_O_18_{(OCH_2_)_3_CNHCOC_6_H_5_}_2_] (**Na-FeMo_6_-bzn**), Na_3_[FeMo_6_O_18_{(OCH_2_)_3_CNHCOC_8_H_7_}_2_] (**Na-FeMo_6_-cin**), Na_3_[MnMo_6_O_18_{(OCH_2_)_3_CNHCOC_6_H_5_}_2_] (**Na-MnMo_6_-bzn**), and Na_3_[MnMo_6_O_18_{(OCH_2_)_3_CNHCOC_8_H_7_}_2_] (**Na-MnMo_6_-cin**; Figures S2 and S5 in the Supporting Information). The final water solubility was 80 mm for **Na-FeMo_6_-bzn** and **Na-MnMo_6_-bzn** and 40 mm for **Na-FeMo_6_-cin** and **Na-MnMo_6_-cin** with the larger hydrophobic cinnamic acid ligand.

### X-ray structural characterization

**Figure 1:**
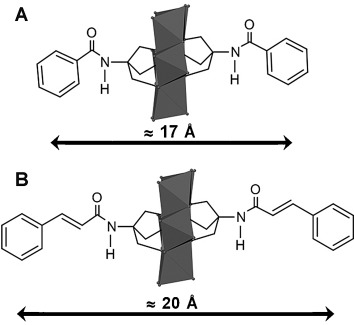
Combined skeletal/polyhedral representation of A) [XMo_6_O_18_{(OCH_2_)_3_CNHCOC_6_H_5_}_2_]^3−^ (X=Fe^3+^ (TBA-FeMo_6_-bzn), Mn^3+^ (TBA-MnMo_6_-bzn)) and B) [XMo_6_O_18_{(OCH_2_)_3_CNHCOC_8_H_7_}_2_]^3−^ (X=Fe^3+^ (TBA-FeMo_6_-cin), Mn^3+^ (TBA-MnMo_6_-cin)). Legend: MoO_6_: grey octahedra; O: grey spheres. Hydrogen atoms are omitted for clarity.

X-ray crystallographic analysis shows that the asymmetric units in **TBA-FeMo_6_-bzn**, **TBA-FeMo_6_-cin**, **TBA-MnMo_6_-bzn**, and **TBA-MnMo_6_-cin** consist of the hybrid Anderson POM (Figure 1), three TBA counterions, and ACN solvent molecules (2.5 to 3.5). A summary of crystal parameters and refinement details are shown in Table 1. The structural analysis revealed that all four compounds crystallize in the monoclinic crystal system, space group *C*2/*c* for **TBA-FeMo_6_-bzn** and **TBA-MnMo_6_-bzn** whereas **TBA-FeMo_6_-cin** and **TBA-MnMo_6_-cin** crystallize in space group *P*2_1_/*n*.

**Table 1:** Crystallographic data for TBA-FeMo_6_-bzn, TBA-FeMo_6_-cin, TBA-MnMo_6_-bzn, and TBA-MnMo_6_-cin.

	TBA-FeMo_6_-bzn	TBA-FeMo_6_-cin	TBA-MnMo_6_-bzn	TBA-MnMo_6_-cin
formula	C_77_H_142.5_FeMo_6_N_8.5_O_26_	C_79_H_143.50_FeMo_6_N_7.50_O_26_	C_77_H_142.5_MnMo_6_N_8.5_O_26_	C_79_H_143.5_MnMo_6_N_7.5_O_26_
*m*_r_ [g cm^−3^]	2234.98	2245.99	2234.07	2243.07
space group	*C*2/*c*	*P*2_1_/*n*	*C*2/*c*	*P*2_1_/*n*
crystal system	monoclinic	monoclinic	monoclinic	monoclinic
*a* [Å]	27.0330(16)	14.8218(9)	27.0398(19)	14.8671(10)
*b* [Å]	26.8760(16)	27.4955(18)	26.9078(19)	27.4468(19)
*c* [Å]	27.6294(18)	23.9333(14)	27.6186(19)	23.8225(16)
*α* [°]	90	90	90	90
*β* [°]	105.084(3)	92.3575(14)	104.729(3)	92.404(2)
*γ* [°]	90	90	90	90
*V* [Å^3^]	19 382(2)	9745.3(10)	19 434(2)	9712.3(11)
*Z*	8	4	8	4
*μ* [mm^−1^]	0.969	0.964	0.947	0.948
reflns. collected	221 627	126 892	378 613	268 583
indep. reflns.	17 745	28 975	17 792	28 596
*R*_int_	0.0714	0.0699	0.0718	0.0687
GOF on *F*^2^	1.123	1.009	1.085	1.031
*R*_1_ [*I*>2*σ*(*I*)]	0.0517	0.0484	0.0317	0.0458
*wR*_2_ (all data)	0.1337	0.1185	0.0850	0.1114

All four compounds show the characteristic Anderson-type structure with a central XO_6_ (X=Mn^3+^, Fe^3+^) octahedron surrounded by six edge-shared MoO_6_ octahedra that form a planar array of distorted octahedra that originates from the outwards expansion of the Mo atoms, all in agreement with reported Anderson structures.^39^ The central octahedron is also slightly flattened as indicated by the summarized bond lengths in Table 2. Three different coordination modes of oxygen atoms are found in the structure; six triple-bridged oxygen atoms connect the heteroatom and two Mo atoms, six double-bridged oxygen atoms connect two Mo atoms, and two terminal oxygen atoms are connected to each of the six Mo atoms. The bond lengths of the three different binding modes are summarized in Table 2 and are in good agreement with other TRIS-functionalized Anderson POMs.^28^, ^38^ The organic ligands are grafted directly onto the oxygen atoms that surround the heteroatom (Figure 1).

**Table 2:** Selected bond lengths [Å] for the anions in TBA-FeMo_6_-bzn, TBA-FeMo_6_-cin, TBA-MnMo_6_-bzn, and TBA-MnMo_6_-cin; O_t_=terminal oxygen atoms.

	TBA-FeMo_6_-bzn	TBA-FeMo_6_-cin	TBA-MnMo_6_-bzn	TBA-MnMo_6_-cin
Mn/Fe–O	1.980(12)–1.990(11)	1.981(11)–1.999(4)	1.957(4)–2.016(9)	1.911(11)–2.054(12)
Mn/Fe–O–Mo	2.335(14)–2.410(12)	2.350(8)–2.396(12)	2.331(13)–2.378(9)	2.314(12)–2.405(9)
Mo–O–Mo	1.910(9)–1.928(13)	1.914(9)–1.927(11)	1.911(12)–1.928(12)	1.909(15)–1.929(11)
Mo–O_t_	1.699(8)–1.706(12)	1.696(11)–1.709(13)	1.697(15)–1.707(13)	1.700(9)–1.710(10)

**Figure 2:**
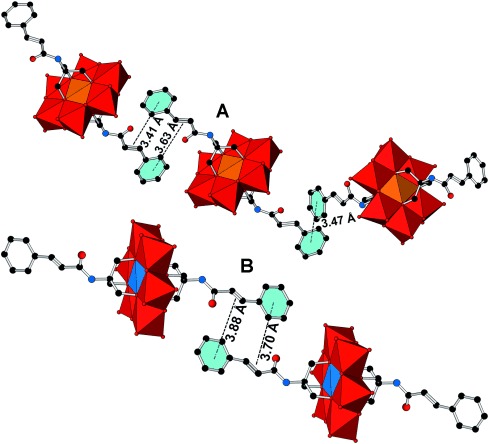
π–π interactions found in A) TBA-FeMo_6_-cin and B) TBA-MnMo_6_-cin and their observed separations in the crystal structures. Legend: MoO_6_: red octahedra; FeO_6_: orange octahedra; MnO_6_: blue octahedra; C: black spheres; N: blue spheres; O: red spheres. Hydrogen atoms are omitted for clarity.

Compounds **TBA-FeMo_6_-cin** and **TBA-MnMo_6_-cin**, which contain the larger cinnamic acid, showed distorted aromatic units and after appropriate consideration (see the Experimental Section in the Supporting Information for details), possible π–π interactions in the crystal structure based on geometry and separation (Figure 2) were found. **TBA-FeMo_6_-cin** displays two different modes of π–π interaction between conjugated systems (Figure 2A), either between two aromatic rings or between the aromatic ring and the π* orbital in C=C. The crystallographic refinement results for **TBA-MnMo_6_-cin** suggest only π–π interactions between the aromatic ring and C=C (Figure 2B) based on geometry and separation. The separations vary between 3.41 and 3.88 Å for all π–π interactions between the aromatic ring and C=C in both compounds, which compares well with previous reports.^40^ The interactions arrange in a parallel offset face-to-face-type π–π stacking. The exact type of stacking between the two aromatic rings is difficult to identify due to the distortion, but is predominantly of a T-shaped character with a separation of 3.47 Å.

Interestingly, TBA-FeMo_6_-bzn and TBA-MnMo_6_-bzn, which contain benzoic acid, do not display any π–π interaction between the aromatic units, possibly due to the shorter carbon chain compared with compounds that contain the cin ligand. Thus, π–π interactions would require two POM units at a close separation, which is electrostatically unfavorable.

Compounds TBA-FeMo_6_-bzn and TBA-MnMo_6_-bzn show similar crystal packing due to the common organic ligand, which consists of alternate layers of TBA counterions and ACN solvent molecules and alternate layers of the hybrid POM. The alternate layers are repeated along the *b* plane. Compounds TBA-FeMo_6_-cin and TBA-MnMo_6_-cin also show similar crystal packing and consist of alternate layers. The first layer is comprised of the inorganic POM and TBA cations and the second layer are composed of the organic ligand grafted onto the POM and TBA cations. This build up is found along both the *a* and *c* planes.

### FTIR spectroscopy

The IR transmission spectra of **TBA-FeMo_6_-bzn**, **TBA-FeMo_6_-cin**, **TBA-MnMo_6_-bzn**, and **TBA-MnMo_6_-cin** are presented and discussed in the Supporting Information (Figure S1).

### ESI-MS characterization and hydrolytic stability study

Electrospray ionization mass spectrometry (ESI-MS) was used to characterize **TBA-FeMo_6_-bzn**, **TBA-FeMo_6_-cin**, **TBA-MnMo_6_-bzn**, and **TBA-MnMo_6_-cin**. The most relevant peak envelopes of (TBA)[FeMo_6_O_18_(C_22_H_24_N_2_O_8_)]^2−^ (calcd: 803.4; found: 803.4), (TBA)[FeMo_6_O_18_(C_26_H_28_N_2_O_8_)]^2−^ (calcd: 829.4; found: 829.4), (TBA)[MnMo_6_O_18_(C_22_H_24_N_2_O_8_)]^2−^ (calcd: 802.9; found: 802.9), and (TBA)[MnMo_6_O_18_(C_26_H_28_N_2_O_8_)]^2−^ (calcd: 828.9; found: 828.9), which confirmed the presence of the intact clusters in the compounds, are shown in Figure S6 in the Supporting Information. The rest of the spectra display a quite complex fragmentation pattern but are similar for all four compounds. They form mainly oxo-molybdo fragments with Mo in different oxidation states, in accordance with previous reports.^41^

The hydrolytic stability of compounds **Na-FeMo_6_-cin** and **Na-MnMo_6_-bzn** were subject to investigation with ESI-MS after 24 h in aqueous buffer solutions in the pH range of 4 to 9. Reports on hydrolytic stability are rather scare due to the challenge of maintaining the correct isomer in solution or preventing conversion into different structures.^42^ POMs that have been organically modified and feature both covalent and noncovalent attachment of organic ligands and biomolecules have all shown increased hydrolytic stability under physiological conditions.^43^, ^44^ Recently, the single-side grafted [GaMo_6_O_18_(OH)_3_{(OCH_2_)_3_CCH_2_OH}]^3−^ anion was also confirmed to be stable in the pH range of 4 to 9 for up to 24 h.^24^

**Figure 3:**
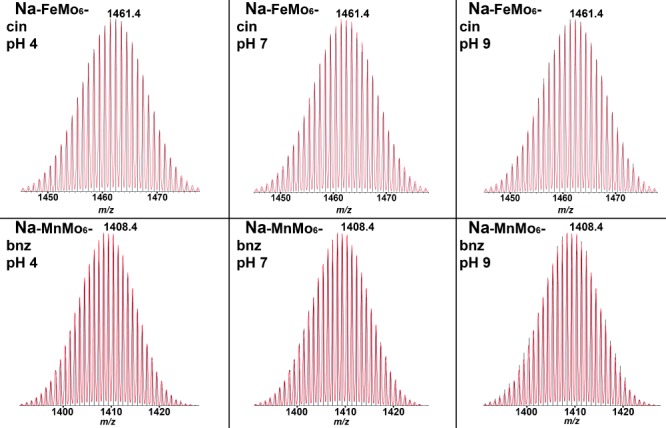
Peak envelopes of Na-FeMo_6_-cin (top) and Na-MnMo_6_-bzn (bottom) at pH 4 (left), 7 (center), and 9 (right) after 24 h; experimental pattern is in black and simulated pattern is overlaid in red.

Samples of **Na-FeMo_6_-cin** and **Na-MnMo_6_-bzn** were dissolved in water that contained 10 mm buffers at pH 4 (ammonium acetate), 7 (ammonium bicarbonate), and 9 (ammonium carbonate) and ESI-MS spectra were recorded after 24 h. Figure 3 shows peak envelopes of the intact clusters Na_2_[FeMo_6_O_18_{(OCH_3_CNHCOC_8_H_7_}_2_]^−^ (found: 1461.4; calcd: 1461.4) and Na_2_[MnMo_6_O_18_{(OCH_2_)_3_CNHCOC_6_H_5_}_2_]^−^ (found: 1408.4; calcd: 1408.4) at the three different pH values. This qualitatively confirms the presence of the intact clusters, with a satisfactory overlap of the superimposed simulated pattern. The full spectra are highly similar in terms of fragmentation pattern and the relative intensity of the main peaks in all samples in the pH study (Figure 3). The spectra are also similar to **TBA-FeMo_6_-cin** and **TBA-MnMo_6_-bzn** recorded in the absence of any buffer (Figure S6 in the Supporting Information). This indicates a comparable POM concentration in all samples and suggests solution stability over a pH range from 4 to 9.

### SDS-PAGE study

SDS-PAGE was applied to show that **Na-FeMo_6_-bzn**, **Na-FeMo_6_-cin**, **Na-MnMo_6_-bzn**, and **Na-MnMo_6_-cin** are hydrolytically inactive towards proteins. Hydrolytic activity has been reported for POM archetypes in which the heteroatoms are more exposed.^15^ Human and bovine serum albumin (HSA and BSA) proteins were chosen because their positive surface charge has been extensively investigated under the typical physical conditions applied in protein crystallization. The aqueous buffer systems used are the same as in the hydrolytic stability study performed by using ESI-MS. Compounds **Na-FeMo_6_-bzn**, **Na-FeMo_6_-cin**, **Na-MnMo_6_-bzn**, and **Na-MnMo_6_-cin** were added in 10- and 100-fold excess of HSA/BSA and were analyzed after 4 d at 20 °C. Controls with starting reagents and nondecorated Anderson POMs are included in the experiment. The SDS-PAGE (14 % polyacrylamide gel) was stopped just before the loading buffer finished traveling across the gel to insure detection of small protein fractions.

**Figure 4:**
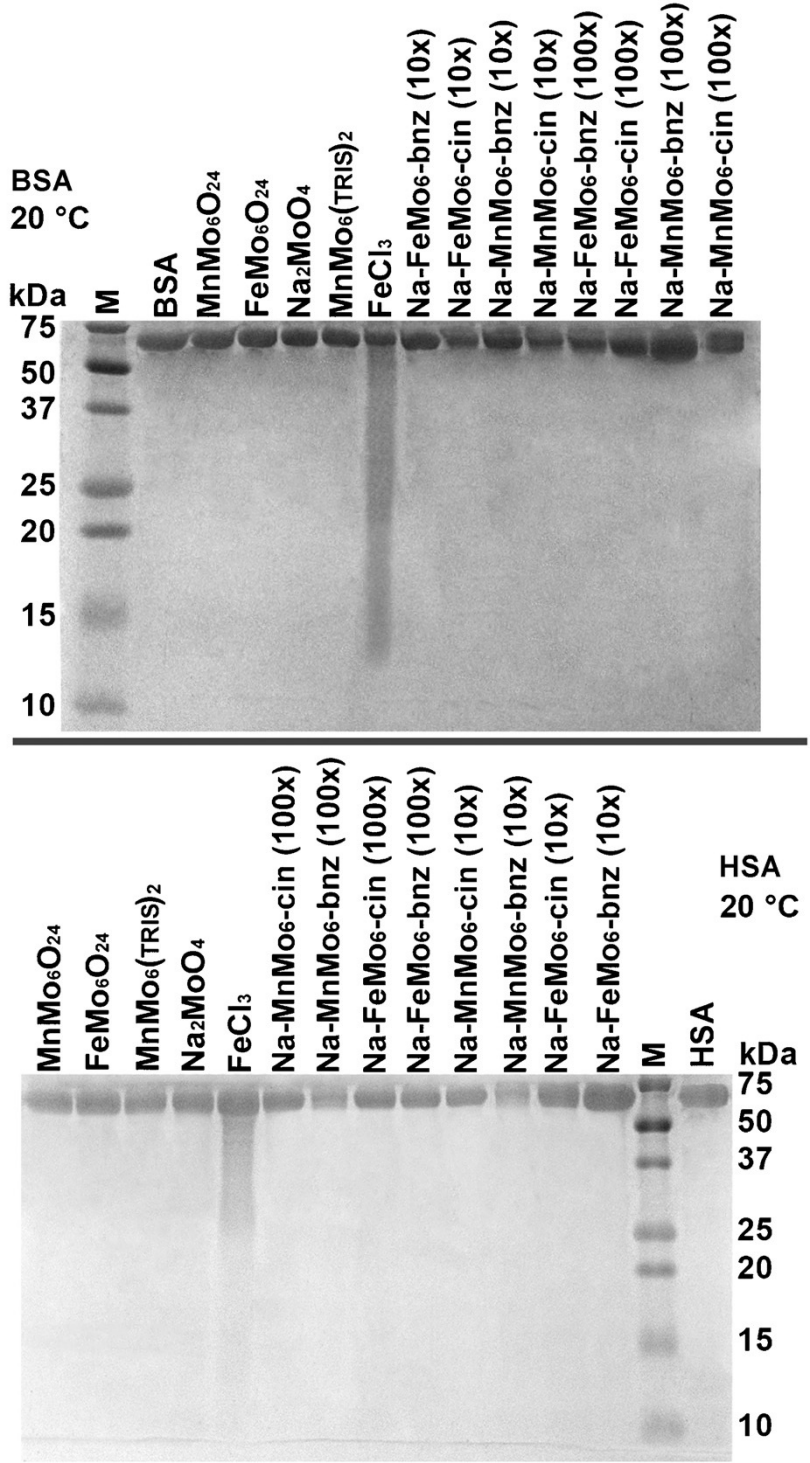
SDS-PAGE screening of BSA/HSA solutions that contain Na-FeMo_6_-bzn, Na-FeMo_6_-cin, Na-MnMo_6_-bzn, and Na-MnMo_6_-cin in 10 and 100-fold excess. Reagents used to synthesize the POMs are also included in 100-fold excess. MnMo_6_O_24_=Na_3_[Mn(OH)_6_Mo_6_O_18_]; FeMo_6_O_24_=Na_3_[Fe(OH)_6_Mo_6_O_18_]; MnMo_6_O_24_(TRIS)_2_=TBA_3_[FeMo_6_O_18_{(OCH_2_)_3_CNH_2_}_2_];^27^ M=Marker. Top: SDS-PAGE samples with BSA; bottom: SDS-PAGE samples with HSA.

Both gels show (Figure 4) the intact serum albumin protein at 66 (BSA) or 66.5 kDa (HSA) with no lower mass fractions detectable, even at a 100-fold excess of **Na-FeMo_6_-bzn**, **Na-FeMo_6_-cin**, **Na-MnMo_6_-bzn**, and **Na-MnMo_6_-cin**, in agreement with previously reported TRIS-functionalized Anderson POMs.^24^ The only sample that showed lower mass fractions was that with FeCl_3_, which indicates nonspecific cleavage with both proteins due to the Lewis acid properties of Fe^3+^.^45^

### Fluorescence quenching measurements to investigate the POM–HSA interaction

Fluorescence quenching is a well-established experiment for the investigation of ligand–protein interactions. Therefore, it has been previously used to analyze the interactions between HSA/BSA and different POMs, including Keggin, Wells–Dawson, Lindqvist, and wheel-shape-structured POMs.^46^–^50^ However, all of these investigations were performed with solely inorganic POMs. Herein, fluorescence quenching was used to gain more insights into the interaction between aromatic hybrid POMs and the fluorophore tryptophan of HSA. HSA contains one tryptophan residue at position 214, whereas BSA has two that are located at positions 134 and 213 in the amino acid sequence.^51^ HSA and BSA protein were investigated at pH 5.5 and 7.4 in solutions with different concentrations of **Na-FeMo_6_-bzn**, **Na-FeMo_6_-cin**, **Na-MnMo_6_-bzn**, and **Na-MnMo_6_-cin**. The concentration of the proteins was kept constant (1 mg mL^−1^), whereas the POM concentrations were increased up to 0.4-fold (0.006, 0.012, 0.025, 0.05, 0.1, 0.2, and 0.4-fold).

**Figure 5:**
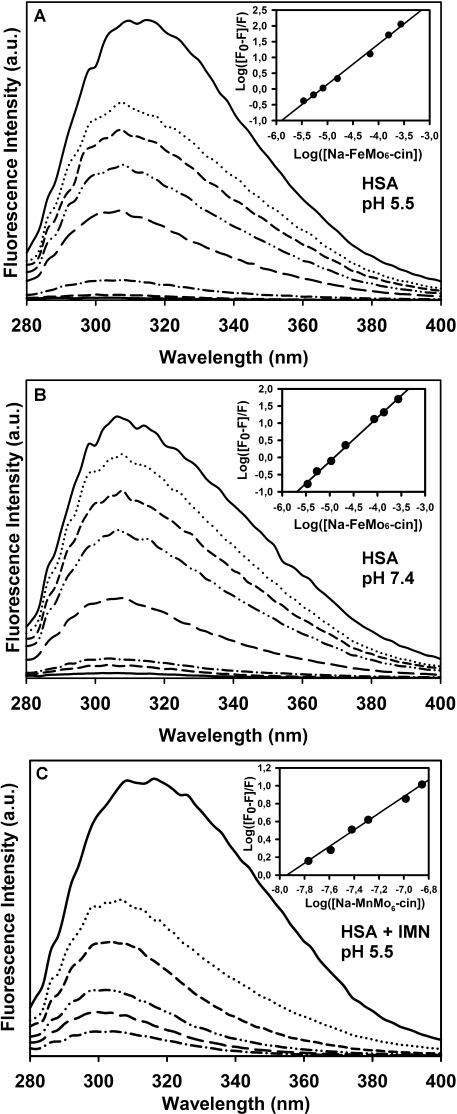
Emission fluorescence spectra of Na-FeMo_6_-cin with HSA ([HSA]=10^−5^ m^−1^) in 10 mm NaOAc buffer at pH 5.5 (A) and 7.4 (B). The top line in each spectrum was recorded in the absence of Na-FeMo_6_-cin followed by stepwise increase (0.006, 0.012, 0.025, 0.05, 0.1, 0.2, and 0.4-fold) of Na-FeMo_6_-cin. The emission fluorescence spectrum in C shows the measurement with indometacin present (0.4-fold) and stepwise Na-FeMo_6_-cin increase (0, 0.1, 0.2, 0.4, 0.8, and 1-fold). Insets: The plot of the derived Stern–Volmer equation (with *R*_2_=0.99).

Table 3 shows the calculated quenching constants for HSA (see Table S1 in the Supporting Information for the BSA data) and the number of bound molecules. Figure 5A, B shows the emission spectra and corresponding derived Stern–Volmer plot of **Na-FeMo_6_-cin** (the remaining emission and Stern–Volmer plots for **Na-FeMo_6_-cin** and BSA, **Na-FeMo_6_-bzn**, **Na-MnMo_6_-bzn**, and **Na-MnMo_6_-cin** with BSA and HAS are given in Figures S7–S10 in the Supporting Information).

**Table 3:** Quenching constants and number of binding molecules for the investigated albumin proteins and pH values.

POM	Protein	*K*_q_ [m^−1^]	*n*	pH
**Na-FeMo_6_-bzn**	HSA	1.3×10^5^	1.3	5.5
**Na-FeMo_6_-cin**	HSA	1.4×10^5^	1.2	5.5
**Na-MnMo_6_-bzn**	HSA	4.4×10^5^	1.3	5.5
**Na-MnMo_6_-cin**	HSA	1.2×10^5^	1.3	5.5
**Na-FeMo_6_-bzn**	HSA	5.0×10^4^	1.1	7.4
**Na-FeMo_6_-cin**	HSA	9.3×10^4^	1.2	7.4
**Na-MnMo_6_-bzn**	HSA	6.0×10^4^	1.1	7.4
**Na-MnMo_6_-cin**	HSA	9.8×10^4^	1.2	7.4
**Na-MnMo_6_-cin**	HSA+IMN	2.5×10^6^	0.9	5.5

The emission spectra show a maximum at *λ*=312 nm, which decreases with increasing POM concentrations due to binding of POM to HSA. In addition, there is a noticeable shift towards lower wavelengths (with increasing POM concentration), which suggests a decrease in polarity within the immediate environment of the tryptophan. The quenching constants are higher at lower pH (5.5) due to the higher overall surface charge of the protein, which is in accordance with previous results.^50^ It is also worth noting that all POM hybrids form a 1:1 complex with the protein, which is also in agreement with previous reports that involve different inorganic POM archetypes.^46^–^50^ This indicates not only successful POM binding but that the interaction can only take place at a conformational strongly defined site, which was suggested to be subdomain IIA. It is well established that compounds that bind to subdomain IIA are likely to enhance fluorescence quenching of HSA because compounds bound at other cavities (e.g., subdomain IIIA) would not exhibit any fluorescence quenching due to their greater distance from tryptophan 214. Therefore, it is suggested that the aromatic hybrid POMs reported herein interact with subdomain IIA.

To confirm this binding site, further fluorescence quenching experiments were performed with a HSA–indometacin (HSA-IMN) complex. IMN is known to bind to subdomain IIA of HSA, which was proven by X-ray crystallography.^52^ Moreover, it has been shown that IMN is able to bind to this site in the presence of other compounds, such as cinnamic acid. Therefore, several emission fluorescence spectra of HSA-IMN-Na-MnMo_6_-cin were recorded (Figure 5C). The calculated binding constant of HSA-IMN-Na-MnMo_6_-cin was significantly larger than that of HSA-Na-MnMo_6_-cin without the drug (Table 3), which indicates that both the drug and the hybrid POM were simultaneously bound to subdomain IIA of HSA.

**Figure 6:**
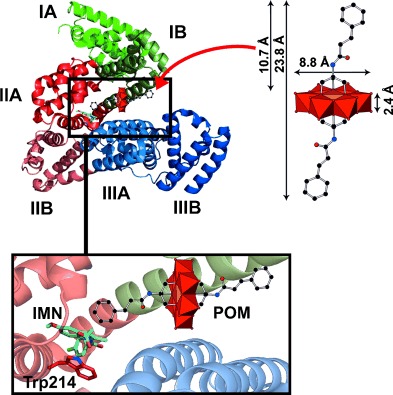
Hypothetical binding of aromatic hybrid POM to HSA. HSA is shown as a ribbon structure with all subdomains represented in different colors. Right: The aromatic hybrid POM is depicted with its dimensions. The red arrow indicates the big cavity that leads to subdomain IIA (in red). Inset: A closer view of the hypothetical position of the POM together with IMN, which is located next to fluorophore Trp214. The POM is illustrated as a combination of polyhedra and sticks, whereas IMN and Trp214 are illustrated sticks (color code: IMN: carbon=cyan, blue=nitrogen, red=oxygen, light green=chloride; Trp214: red=carbon, blue=nitrogen).

Taking into account the size of the hybrid POM **Na-MnMo_6_-cin** (23.8×8.8×2.4 Å) and the sizes of all HSA cavities, there is only one cavity in which the aromatic hybrid POMs are sterically able to penetrate the core of HSA, namely the cavity that leads to subdomain IIA (Figure 6).

With regard to the types of interaction between HSA and the aromatic hybrid POMs, electrostatic and especially hydrophobic interactions are suggested. A control experiment with Na_3_[Fe(OH)_6_Mo_6_O_18_] up to two molar equivalents showed no quenching of the tryptophan signal at either pH value (5.5 and 7.4), which revealed no binding in the vicinity of tryptophan 214 and thus indicated the importance of the aromatic moieties in the binding of the hybrid POMs reported herein. It is suggested that the aromatic hybrid POM approaches subdomain IIA through the above-mentioned cavity and then exhibit hydrophobic interactions with its hydrophobic tails, whereas the Anderson core is stabilized through electrostatic interactions with polar amino acid side chains from, for example, subdomain IB (Figure 6). This is not surprising because the organic moiety of the hybrid POMs are structurally very similar to cinnamic acid, which has been shown to bind to HSA subdomain IIA (together with IMN) by directly interacting with Trp214.^52^ Thus, the aromatic hybrid POMs reported herein might interact similarly with HSA through their organic groups.

## Conclusion

Four different hybrid organic–inorganic Anderson POMs were synthesized in an organic solvent and introduced to aqueous environments through a cation exchange step. They show robust hydrolytic stability in a pH range of 4 to 9 for up to 24 h and are hydrolytically inactive in aqueous buffer solutions in the presence of BSA and HSA proteins. Instead, they interact through electrostatic, hydrophobic, or π–π interactions, or a combination of these. This introduces the possibility for another mode of POM–protein interaction in addition to the demonstrated electrostatic interaction. The terminal oxygen atoms on the Anderson POM can interact electrostatically with positively charged amino acids. The double-sided grafting of the aromatic ligands may allow for π–π interactions or hydrophobic interactions at two different sites. This makes the hybrid Anderson POMs reported herein potentially superior to pure inorganic structures that have been successfully applied so far as additives in macromolecular crystallography. Thus, they may in theory stabilize new protein regions that have not been accessible so far for POMs, which could result in the formation of new protein crystals promoted by POMs.

## Experimental Section

Full experimental data and synthesis procedures can be found in the Supporting Information. All reagents and chemicals were of analytical grade and used without further purification. All reagents and chemicals were supplied by Sigma–Aldrich Chemical Company and solvents were supplied by Merck Chemicals. Single-crystal X-ray diffraction data were collected at 100 K by using a Bruker D8 Venture diffractometer equipped with a multilayer monochromator, a Mo_Kα_ INCOATEC microfocus sealed tube (*λ*=0.71073 Å), and a CMOS Photon Detector. CCDC 1406885 (**TBA-FeMo_6_-bzn**,), 1406886 (**TBA-MnMo_6_-bzn**), 1406887 (**TBA-FeMo_6_-cin**), and 1406888 (**TBA-MnMo_6_-cin**) contain the supplementary crystallographic data for this paper. These data are provided free of charge by The Cambridge Crystallographic Data Centre.

### Synthesis of TBA-FeMo_6_-bzn, (TBA)_3_[FeMo_6_O_18_{(OCH_2_)_3_CNHCOC_6_H_5_}_2_]⋅3.5 ACN

The synthesis was carried out according to a published procedure.^36^ Tetrabutylammonium octamolybdate was dissolved in acetonitrile and heated at reflux with Fe(acac)_3_ and the ligand (HOCH_2_)_3_CNHCOC_6_H_5_ for 18 h. After cooling to RT, the red mixture was centrifuged to remove the precipitate and give a dark red solution. Crystals suitable for X-ray crystallographic analysis were obtained through ether diffusion after a few days. FTIR: $\tilde \nu $
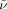
=2960 (v CH_3_, s), 2934 (v CH_3_, s), 2873 (v CH_3_, s), 1674 (v C=O, s), 1599 (v Ar, w) 1578 (v Ar, w), 1517 (v Ar, m), 1482 (δ CH_2_, s), 1380 (δ CH_3_, m), 1319 (m), 1268 (m), 1102 (m), 1031 (v C–O, m), 939 (s), 918 (s), 902 (v Mo=O, s), 808 (w), 647 (v Mo-O-Mo, s), 559 (m) 406 cm^−1^ (m). Elemental analysis calcd (%) for FeMo_6_O_26_C_70_H_132_N_5_ (2091.3 g mol^−1^): C 40.20, H 6.31, O 19.41, N 3.26, Fe 2.67, Mo 28.01; found: C 40.19, H 6.28, O 19.38, N 3.24, Fe 2.61, Mo 27.94.

Synthetic procedures for **TBA-FeMo_6_-bzn**, **TBA-FeMo_6_-cin**, **TBA-MnMo_6_-bzn**, **TBA-MnMo_6_-cin**, **Na-FeMo_6_-bzn**, **Na-FeMo_6_-cin**, **Na-MnMo_6_-bzn**, **Na-MnMo_6_-cin**, bzn, and cin, and the full experimental information are given in the Supporting Information.

## Supporting Information

As a service to our authors and readers, this journal provides supporting information supplied by the authors. Such materials are peer reviewed and may be re-organized for online delivery, but are not copy-edited or typeset. Technical support issues arising from supporting information (other than missing files) should be addressed to the authors.

**miscellaneous_information**
