# Supplementary material for: The Synthesis and Characterization of Aromatic Hybrid Anderson–Evans POMs and their Serum Albumin Interactions: The Shift from Polar to Hydrophobic Interactions
Source: Chemistry. 2015 Nov 3;21(49):17800–7. doi: 10.1002/chem.201502458 (PMC4676910; doi:10.1002/chem.201502458)
Supplement: Supplementary file 1 [file chem0021-17800-sd1.pdf]

CHEMISTRY
A European Journal

Supporting Information

The Synthesis and Characterization of Aromatic Hybrid Anderson–Evans POMs and their Serum Albumin Interactions: The Shift from Polar to Hydrophobic Interactions

Emir Al-Sayed,[a] Amir Blazevic,[a] Alexander Roller,[b] and Annette Rompel*[a]

chem_201502458_sm_miscellaneous_information.pdf

Table of Contents

1. Experimental section S2

2. Synthesis procedure S4

3. FTIR spectroscopy S7

4. ^{1}H-NMR spectroscopy S8

5. ESI-MS S10

6. Tryptophan fluorescence quenching S11

7. References S14

1. Experimental section

Materials and equipment

Elemental Analysis: The determination of C/H/N/O was done using an "EA 1108 CHNS-O" elemental analyzer by Carlo Erba Instruments at the Mikroanalytisches Laboratorium, University of Vienna. **Inductively coupled plasma-optical emission spectrometry:** Elements were determined using Optima 5300DV (Perkin Elmer) in aqueous solutions containing 2 % ultrapure HNO_3. **Electrospray Ionization Mass Spectrometry:** Compounds **TBA−FeMo₆−bnz**, **TBA−FeMo₆−cin**, **TBA−MnMo₆−bnz**, **TBA−MnMo₆−cin**, **Na−FeMo₆−cin** and **Na−MnMo₆−bnz** were investigated with an ESI−Qq−oaRTOF supplied by Bruker Daltonics Ltd. at the Massenspektrometriezentrum, University of Vienna. Bruker Daltonics Data Analysis software was used to analyze the results. All analyses were carried out in 1:1 mixture of water/ACN, collected in negative ion mode and with the spectrometer calibrated with the standard tune−mix to give a precision of *ca.* 5 ppm in the region of *m/z* 300−3000. **Attenuated total reflection Fourier−transform Infrared Spectroscopy:** All spectra were recorded on a Bruker Tensor 27 IR Spectrometer equipped with a single−reflection diamond−ATR unit. Frequencies are given in cm^{-1}, intensities denoted as w = weak, m = medium, s = strong, vs = very strong, sh = sharp, br = broad. **Nuclear magnetic resonance:** NMR spectra were recorded on a Bruker FT-NMR Avance III 500 MHz instrument at 500.32 (^1H) MHz in D_2O at ambient temperatures. Chemical shifts were referenced relative to the solvent signal for ^1H. The splitting of proton resonances in the ^1H NMR spectra are defined as s = singlet, bs = broad singlet, d = doublet, dd = doublet of doublets, ddd = doublet of doublets of doublets, t = triplet, dt = doublet of triplets, and m = multiplets. **SDS−PAGE:** BSA and HSA (1 mg/mL) were dissolved in water with 10 or 100 equivalents of **Na−FeMo₆−bnz**, **Na−FeMo₆−cin**, **Na−MnMo₆−bnz** and **Na−MnMo₆−cin** and incubated for 4 days at 20 °C. SDS–PAGE was performed according to a previously published procedure[1] using Precision Plus Protein Standard Dual Color (Bio−Rad) as molecular weight marker. Samples were applied to 14% polyacrylamide gels mixed with reduced S3 loading dye. Sample load onto the gel was 5 µg. Gels were stained with Coomassie Brilliant Blue. Imaging of the gels was done with Gel Doc™ XR of BIO−RAD. **Tryptophan fluorescence spectroscopy:** **Na−FeMo₆−bnz**, **Na−FeMo₆−cin**, **Na−MnMo₆−bnz, Na−MnMo₆−cin** and Indometacin were recorded

on a Infinite 200 microplate reader using Nunclon 96 Flat Bottom Transparent Polystyrol plates. The range of the emission wavelength was between 280 and 400 nm. The absorbance from the POMs was deducted from the raw quenching data by applying equation [2] and (2).[3]

$$OD = \log\frac{I_0}{I} = \frac{1}{2}\,\varepsilon_{235}CL \qquad 2$$

$$F = F_1\frac{I_0}{I} \qquad (2)$$

The further evaluation of the results was done with a derived Stern−Volmer equation [2].[3]

$$\log[(F_0 - F)/F] = \log K_q + n\log[\mathrm{Q}] \qquad 2$$

where F_0 and F are the fluorescence intensities in the absence and presence of the quencher, respectively, K_q the quenching rate constant of the protein, n the number of bound molecules and [Q] the concentration of the quencher. By plotting $\log[(F_0-F)/F]$ versus log[Q] the quenching constant and the number of bound molecules can be extracted. **SXRD:** The data reduction was performed using APEX2 (Bruker Analytical X-ray systems, Madison, 2004.) software package. The structure solution was executed using SHELXS[4] with the GUI OLEX2[5]. The structure refinement was realized with SHELXL[4] and the GUI`s OLEX2[5]. The aromatic units were distorted and were constrained using several eadp constraints. The crystallographic results were proofed with PLATON[6].

2. Synthesis procedure

Cin, $(HOCH_2)_3CNHCOC_8H_7$: The synthesis was carried out according to a published procedure with different educts.[7] Cinnamic acid (1.08 g, 7.32 mmol) and N-methylmorpholine (0,88 ml, 8.00 mmol) were mixed in THF (40 mL). At 0°C ethylchloroformate (0.76 mL, 8.00 mmol) was added slowly. After stirring for 30 min at 0°C, the formed white precipitate was filtered into a solution of TRIS-NH_2 (0.87 g, 7.32 mmol) and triethylamine (1.12 mL, 8.00 mmol) in DMF (40 mL), which had been stirring for at least 10 min. After stirring for 30 minutes at room temperature, the solution was evaporated to remove THF and DMF. Afterwards the residue was extracted in ethylacetate (150 mL) and washed with water. The solvent was dried over $MgSO_4$ and evaporated to dryness. Since there have been no byproducts formed, or any cinnamic acid left, which was confirmed with NMR, there was no need for purification. Yield: 1.8 g (98 %). ^1H NMR (500.32 MHz, D_2O, 25 °C, TMS): δ = 3.64 (s, 6 H), 3.78 (s, 3 H), 6.45 (d, 1 H), 7.32 (d, 1 H), 7.35-7.57 (m, 5 H) ppm. (Figure S1) Elemental analysis $C_{13}H_{17}O_4N$ (251.28 g mol^{-1}): calcd. C 62.1, H 6.8, O 25.5, N 5.6; found C 59.8, H 6.5, O 25.7, N 5.5.

Bnz, $(HOCH_2)_3CNHCOC_6H_5$: The synthesis was similar to that of $(HOCH_2)_3CNHCOC_8H_7$, except that benzoic acid (0.89 g, 7.32 mmol) was used instead of cinnamic acid. Yield: 1.56 g (95 %) ^1H NMR (500.32 MHz, D_2O, 25 °C, TMS): δ = 3.75 (s, 6 H), 3.98 (s, 3 H), 7.45-7.68 (m, 5 H) ppm. (Figure S2). Elemental analysis $C_{11}H_{15}O_4N$ (225.24 g mol^{-1}): calcd. C 58.6, H 6.7, O 28.4, N 6.2; found C 58.9, H 6.5, O 28.6, N 6.5.

TBA−FeMo$_6$−bnz, $(TBA)_3[FeMo_6O_{18}\{(OCH_2)_3CNHCOC_6H_5\}_2]$ × 3.75 ACN: The synthesis was carried out according to a published procedure.[8] Tetrabutylammonium octamolybdate (0.533 g, 0.279 mmol) was dissolved in acetonitrile (20mL) and refluxed with $Fe(acac)_3$ (0.197 g, 0.56 mmol) and ligand (**bnz**, $HOCH_2)_3CNHCOC_6H_5$ (0.188 g, 0.837 mmol) for 18 h. After cooling down to room temperature, the red mixture was centrifuged to remove the precipitate, which leads to a dark red solution. Crystals suitable for X−ray crystallographic analysis were obtained through ether diffusion after a few days. Yield: 0.5 g (86 % based on Mo) FTIR: 2960 (ν CH_3, s), 2934 (ν CH_3, s), 2873 (ν CH_3, s), 1674 (ν C=O, s), 1599 (ν Ar, w) 1578 (ν Ar, w), 1517 (ν Ar, m), 1482 (δ CH_2, s), 1380 (δ CH_3, m), 1319 (m), 1268 (m), 1102 (m),

1031 (ν C−O, m), 939 (s), 918 (s), 902 (ν Mo=O, s), 808 (w), 647 (ν Mo−O−Mo, s), 559 (m) 406 (m) cm^{-1}. Elemental analysis $FeMo_6O_{26}C_{70}H_{132}N_5$ (2091.3 g mol^{-1}): calcd. C 40.20, H 6.31, O 19.41, N 3.26, Fe 2.39, Mo 27.26 ; found C 40.19, H 6.28, O 19.38, N 3.24, Fe 2.36, Mo 26.87.

TBA−FeMo$_6$−cin, (TBA)$_3$[FeMo$_6$O$_{18}${(OCH$_2$)$_3$CNHCOC$_8$H$_7$}$_2$] × 2.5 ACN: The synthesis was similar to that of **TBA−FeMo$_6$−bnz**, except that (**cin**, $HOCH_2)_3CNHCOC_8H_7$ (0.21 g, 0.837 mmol) was used instead of $(HOCH_2)_3CNHCOC_6H_5$. Yield: 0.45 g (75 % based on Mo). FTIR: 2960 (ν CH_3, s), 2939 (ν CH_3, s), 2873 (ν CH_3, s), 1654 (ν C=O, m), 1613 (ν Ar, m) 1549 (ν Ar, m), 1481 (δ CH_2, m), 1380 (δ CH_3, m), 1340 (m), 1285 (m), 1222 (m), 1116 (m), 1020 (ν C-O, s), 978 (ν CH=CH, m), 937 (s), 914 (s), 898 (ν Mo=O, s), 813 (w), 650 (ν Mo-O-Mo, s), 556 (m) 411 (m) cm^{-1}. Elemental analysis $FeMo_6O_{26}C_{74}H_{136}N_5$ (2143.3 g mol^{-1}): calcd. C 41.43, H 6.35, O 19.41, N 3.26, Fe 2.61, Mo 26.88; found C 41.41, H 6.33, O 19.45, N 3.22 Fe 2.58 Mo 26.4.

TBA−MnMo$_6$−bnz, (TBA)$_3$[MnMo$_6$O$_{18}${(OCH$_2$)$_3$CNHCOC$_6$H$_5$}$_2$] × 3.5 ACN: The synthesis was similar to that of **TBA−FeMo$_6$−bnz**, except that $Mn(OAc)_3$ (0.13 g, 0.56 mmol) was used instead of $Fe(acac)_3$. Yield: 0.5 g (86 % based on Mo) ^1H NMR (500.32 MHz, [D_6] DMSO, 25 °C, TMS): δ = 0.93 (t, 36 H), δ = 1.30 (m. 24 H), δ = 1.56 (m, 24 H), δ = 3.15 (m, 24 H), δ = 7.44-7.76 (m, 10 H), δ = 64.5 (s, 12 H) ppm FTIR: 2960 (ν CH_3, s), 2934 (ν CH_3, s), 2873 (ν CH_3, s), 1673 (ν C=O, s), 1600 (ν Ar, w) 1580 (ν Ar, w), 1519 (ν Ar, m), 1482 (δ CH_2, s), 1380 (δ CH_3, m), 1319 (m), 1269 (w), 1152 (m), 1021 (ν C-O, m), 940 (s), 919 (s), 903 (ν Mo=O, s), 810 (w), 648 (ν Mo-O-Mo, s), 561 (m) 411 (m) cm^{-1}. Elemental analysis $MnMo_6O_{26}C_{70}H_{132}N_5$ (2091.3 g mol^{-1}): calcd. C 40.20, H 6.31, O 19.41, N 3.26, Mn 2.39, Mo 27.26; found C 40.15, H 6.25, O 19.39, N 3.22, Mn 2.33, Mo 27.26.

TBA−MnMo$_6$−cin, (TBA)$_3$[MnMo$_6$O$_{18}${(OCH$_2$)$_3$CNHCOC$_8$H$_7$}$_2$] × 2.5 ACN: The synthesis was similar to that of **TBA−FeMo$_6$−cin**, except that $Mn(OAc)_3$ (0.13 g, 0.56 mmol) was used instead of $Fe(acac)_3$. After few days orange crystals were formed and isolated. Yield: 0.4 g (67 % based on Mo) ^1H NMR (500.32 MHz, [D_6] DMSO, 25 °C, TMS): δ = 0.93 (t, 36 H), δ = 1.31 (m. 24 H), δ = 1.57 (m, 24 H), δ = 3.16 (m, 24 H), δ = 6.86 (d, 2 H), δ = 7.24 (d, 2 H) δ = 7.32-7.52 (m, 10 H), δ = 64 (s, 12 H) ppm; FTIR: 2960 (ν CH_3, s), 2936 (ν CH_3, s), 2873 (ν CH_3, s), 1673 (ν C=O, m), 1626 (ν Ar,

m) 1554 (ν Ar, m), 1481 (δ CH_2, s), 1380 (δ CH_3, m), 1342 (m), 1284 (w), 1221 (m), 1113 (m), 1023 (ν C-O, s), 979 (ν CH=CH, m), 938 (s), 914 (s), 898 (ν Mo=O, s), 814 (w), 647 (ν Mo-O-Mo, s), 558 (m) 411 (m) cm^{-1}. Elemental analysis $MnMo_6O_{26}C_{74}H_{136}N_5$ (2143.3 g mol^{-1}): calcd. C 41.43, H 6.35, O 19.41, N 3.26, Mn 2.61, Mo 26.88; found C 41.45, H 6.35, O 19.88, N 3.74, Mn 2.54, Mo 26.88.

Na−$FeMo_6$−bnz, **$Na_3[FeMo_6O_{18}\{(OCH_2)_3CNHCOC_6H_5\}_2]$:** The synthesis was carried out according to a published procedure.[9] **TBA−$FeMo_6$−bnz** (1 g, 0.47 mmol) was dissolved in acetonitrile (20 mL) and added dropwise into a vigorously stirring acetonitrile solution of $NaClO_4$ (1.25 g, 1 mmol), and then the mixed solution was kept stirring for 30 min to obtain an orange precipitate, which was washed twice with acetonitrile. Yield: 0.3 g (45 %). Elemental analysis $Na_3FeMo_6O_{26}C_{22}H_{24}N_2$ (1432.88 g mol^{-1}): calcd. C 18.4, H 1.7, O 29.0, N 1.9, Fe 3.9, Mo 40.2, Na 4.8; found C 18.1, H 1.9, O 29.3, N 1.8, Fe 3.7, Mo 40.0, Na 4.9.

Na−$FeMo_6$−cin, **$Na_3[FeMo_6O_{18}\{(OCH_2)_3CNHCOC_8H_7\}_2]$:** The synthesis was similar to that of **Na−$FeMo_6$−bnz**, except that **TBA−$FeMo_6$−cin** (1 g, 0.67 mmol) was used. Yield: 0.4 g (40 %). Elemental analysis $Na_3FeMo_6O_{26}C_{26}H_{28}N_2$ (1484.95 g mol^{-1}): calcd. C 21.0, H 1.9, O 28.0, N 1.9, Fe 3.8 Mo 38.8 Na 4.6; found C 21.3, H 1.8, O 28.2, N 1.7, Fe 3.9 Mo 38.5 Na 4.4.

Na−$MnMo_6$−bnz, **$Na_3[MnMo_6O_{18}\{(OCH_2)_3CNHCOC_6H_5\}_2]$:** The synthesis was similar to that of **Na−$FeMo_6$−bnz**, except that **TBA−$MnMo_6$−bnz** (1 g, 0.47 mmol) was used. ^1H NMR (500.32 MHz, [D_6]DMSO, 25 °C, TMS): δ = 7.44-7.76 (br, 10 H), δ = 64.5 (s, 12 H) ppm. Yield: 0.35 g (53 %) Elemental analysis $Na_3MnMo_6O_{26}C_{22}H_{24}N_2$ (1431.97 g mol^{-1}): calcd. C 18.4, H 1.7, O 29.0, N 1.9, Mn 3.9, Mo 40.2, Na 4.8; found C 18.2, H 1.9, O 29.2, N 1.6, Mn 3.8, Mo 40.0, Na 4.5.

Na−$MnMo_6$−cin, **$Na_3[MnMo_6O_{18}\{(OCH_2)_3CNHCOC_8H_7\}_2]$:** The synthesis was similar to that of **Na−$FeMo_6$−bnz**, except that **TBA−$MnMo_6$−cin** (1 g, 0.67 mmol) was used. ^1H NMR (500.32 MHz, [D_6]DMSO, 25 °C, TMS): δ = 6.86 (br, 2 H), δ = 7.24 (d, 2 H) δ = 7.32-7.52 (m, 10 H), δ = 64 (s, 12 H) ppm. Yield: 0.45 g (45 %) Elemental analysis $Na_3MnMo_6O_{26}C_{26}H_{28}N_2$ (1484.04 g mol^{-1}): calcd. C 21.0, H 1.9, O 28.0, N 1.9, Mn 3.8 Mo 38.8 Na 4.6; found C 21.3, H 1.7, O 28.2, N 1.6, Mn 3.5 Mo 38.7 Na 4.3.

3. FTIR spectroscopy

In **Figure S1**, the IR transmission spectra of **TBA−FeMo$_6$−bzn**, **TBA−FeMo$_6$−cin**, **TBA−MnMo$_6$−bzn** and **TBA−MnMo$_6$−cin** are presented. The characteristic bridging Mo−O−Mo and terminal Mo=O vibrations appear at 647 cm^{-1} and 920 cm^{-1}, respectively. This confirms the structure of the Anderson POM clusters, regardless of the type of trivalent atom in the center.[10] The attachment of TRIS-bnz and TRIS-cin to the POM is confirmed by bands at 1100 cm^{-1}, which can be assigned to C−O vibrations. Additional vibrations, which characterize the amide vibrations, are found between 1221−1342 cm^{-1}. The carbonyl C=O vibration is present at 1670 cm^{-1} in all four compounds. The vibrations of the CH_3 and CH_2 groups appear above 2873 cm^{-1} and can be assigned to the counter cation TBA.

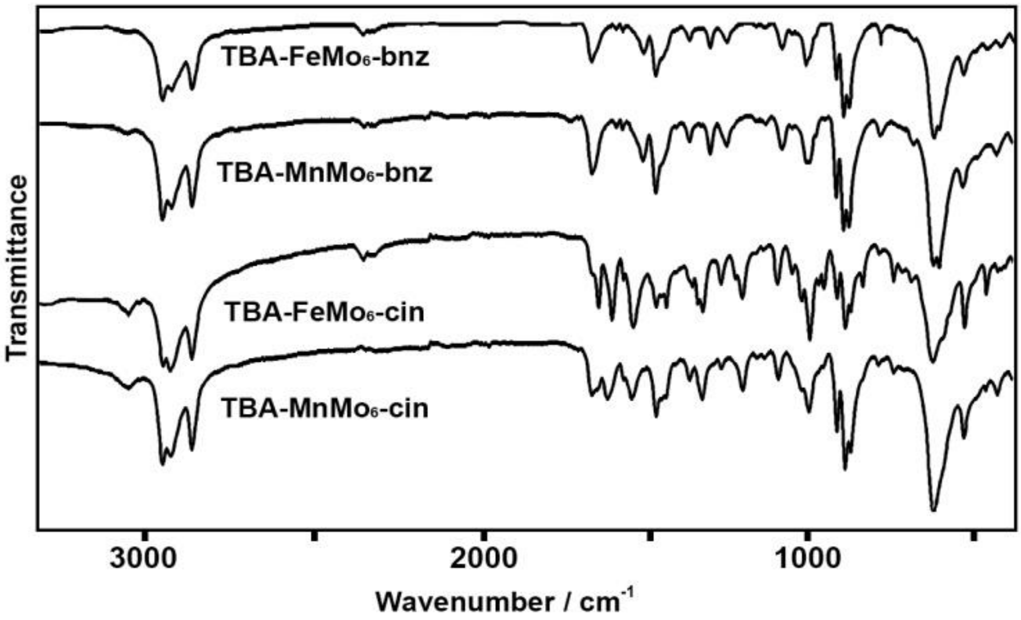

Figure S1. IR spectra comparison of **TBA−FeMo$_6$−bzn**, **TBA−FeMo$_6$−cin**, **TBA−MnMo$_6$−bzn** and **TBA−MnMo$_6$−cin** showing the typical vibrations for Mo=O and Mo–O–Mo and the aromatic organic part independent of central heteroatom (vibrations are listed in the experimental section for all four compounds).

4. NMR spectroscopy

^1H−NMR spectroscopic analysis was used to identify compounds **TBA−MnMo$_6$−bnz**, **TBA−MnMo$_6$−cin**, **Na−MnMo$_6$−bnz** and **Na−MnMo$_6$−cin** in solution and proof successful cation exchange. In **Figure S2** the NMR spectra of compounds **TBA−MnMo$_6$−cin** and **Na−MnMo$_6$−cin** are shown overlapped indicating successful cation exchange from TBA to Na^+ counter cations. The upper spectrum displays the ^1H−NMR signals of **TBA−MnMo$_6$−cin** clearly showing the signals of the aliphatic protons (A, B, C and D) which are lacking in the lower spectrum after ion exchange to Na^+. The olefinic protons F and G appear at similar positions. In the inset, the characteristic shift around 65 ppm of the 12 protons next to the quaternary carbon atom is shown confirming the successful grafting of the organic ligands onto the planar surface of the Anderson POM. NMR spectra of compounds **cin, bzn TBA−MnMo$_6$−bnz** and **Na−MnMo$_6$−bnz** are given in **Figure S3 to S5**.

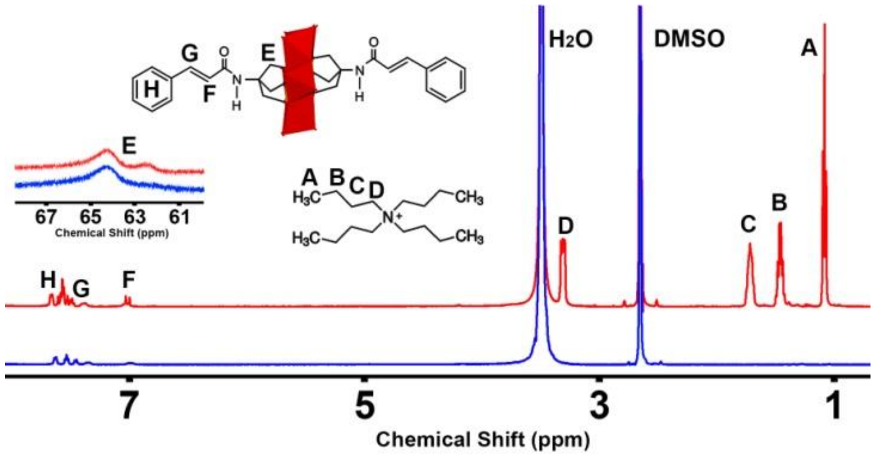

Figure S2. ^1H−NMR spectrum of **Na−MnMo$_6$−cin** (blue) and **TBA−MnMo$_6$−cin** (red) illustrating a successful cation exchange.

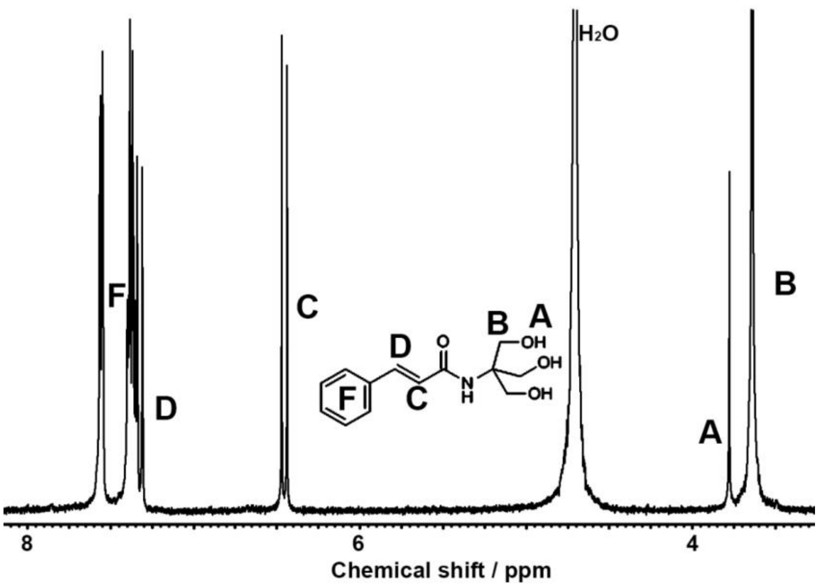

Figure S3. ^{1}H NMR spectrum of **cin**.

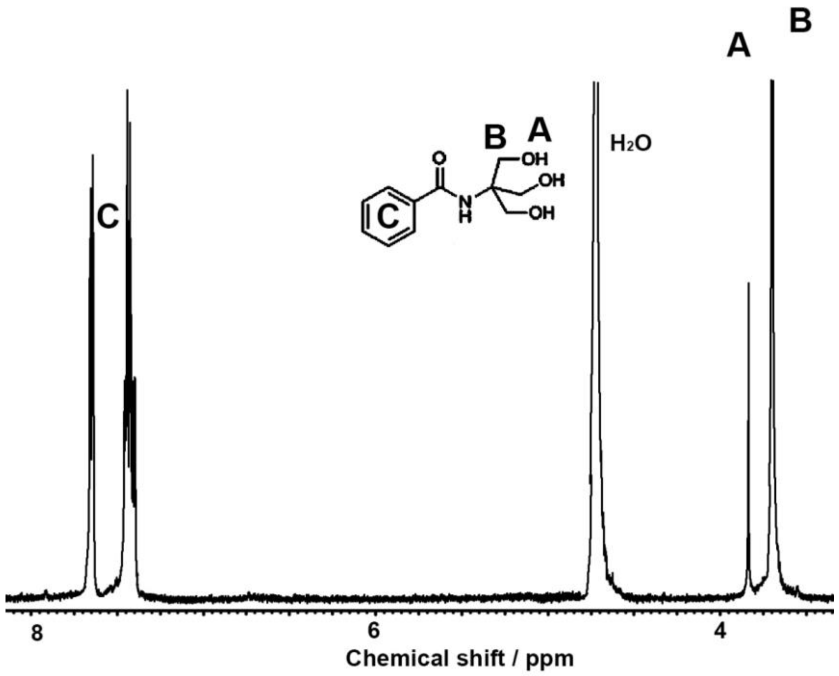

Figure S4. ^{1}H NMR spectrum of **bnz**.

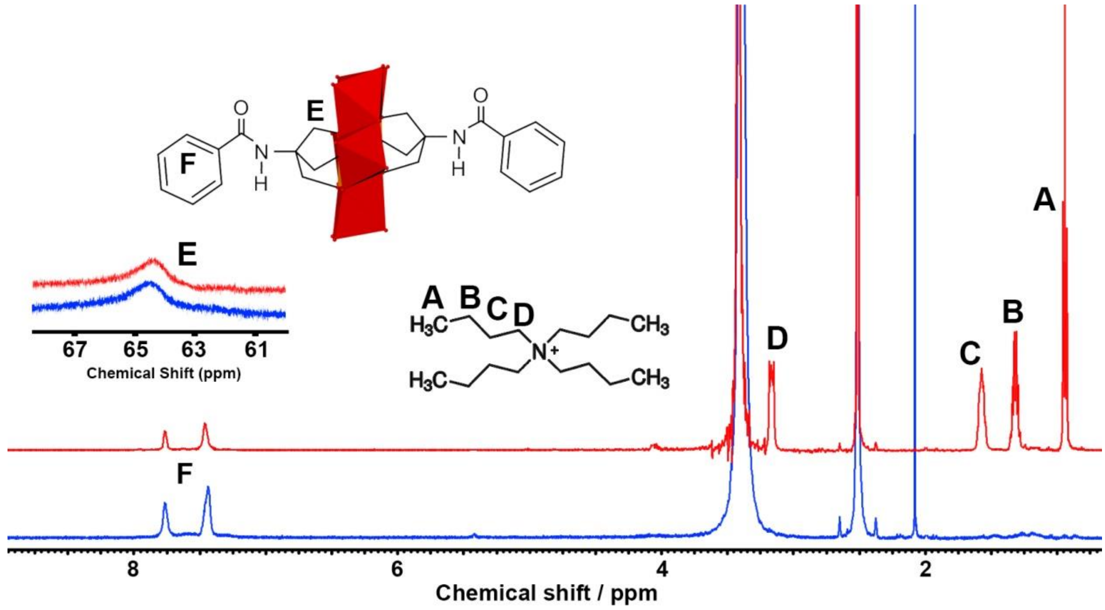

Figure S5. ¹H NMR spectrum of **Na−MnMo$_6$−bnz** (blue) and **TBA−MnMo$_6$−cin** (red).

5. ESI-MS

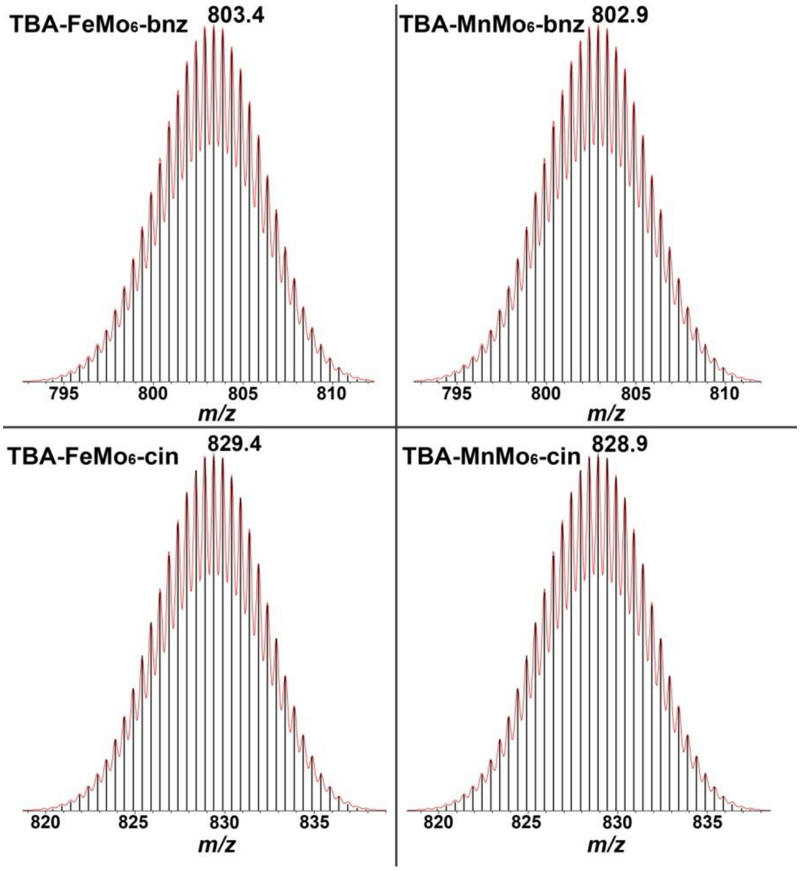

Figure S6. ESI−MS peak envelopes of **TBA−FeMo$_6$−bnz**, **TBA−FeMo$_6$−cin**, **TBA−MnMo$_6$−bnz** and **TBA−MnMo$_6$−cin** with the simulated pattern overlaid in red.

6. Tryptophan fluorescence quenching

Table S1. Quenching constants and number of binding molecules for the BSA protein at pH 5.5 and 7.4.

POM	Protein	K_q (M^{-1})	n	pH
Na−FeMo$_6$−bzn	BSA	3.5×10^5	1.3	5.5
Na−FeMo$_6$−cin	BSA	1.2×10^6	1.4	5.5
Na−MnMo$_6$−bzn	BSA	6.4×10^5	1.3	5.5
Na−MnMo$_6$−cin	BSA	7.5×10^5	1.3	5.5
Na−FeMo$_6$−bzn	BSA	9.4×10^4	1.1	7.4
Na−FeMo$_6$−cin	BSA	9.5×10^4	1.2	7.4
Na−MnMo$_6$−bzn	BSA	2.2×10^5	1.4	7.4
Na−MnMo$_6$−cin	BSA	1.0×10^5	1.3	7.4

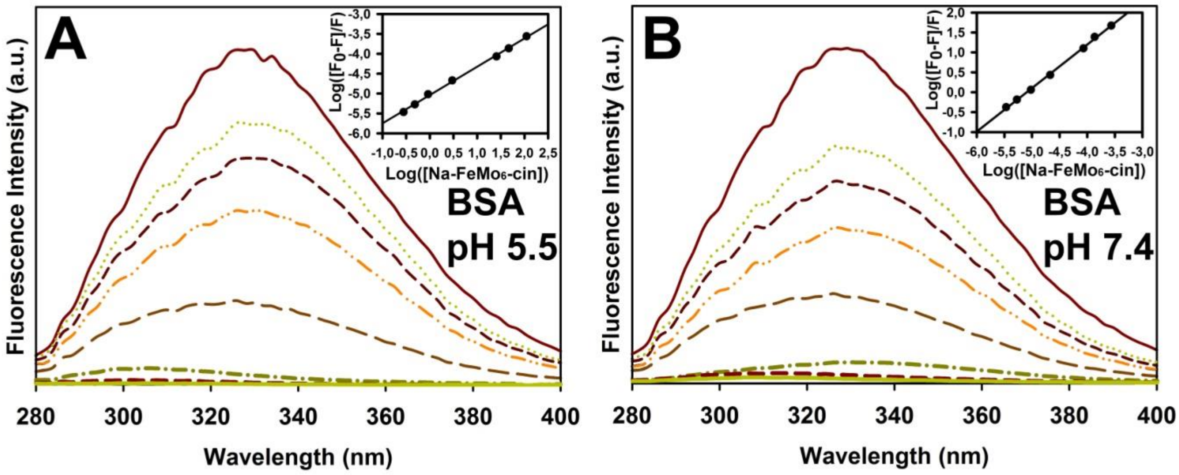

Figure S7. Emission fluorescence spectra of **Na−FeMo$_6$−cin** with BSA [BSA] = 10^{-5} M^{-1}, in 10 mM NaOAc buffer. A: BSA pH 5.5; B: BSA pH 7.4. The top line in each spectrum is collected in the absence of **Na−FeMo$_6$−cin** followed by stepwise increase (0.006, 0.012, 0.025, 0.05, 0.1, 0.2 and 0.4 fold of **Na−FeMo$_6$−cin**). In the inset, the plot of the derived Stern–Volmer equation is depicted (with R_2 = 0.99).

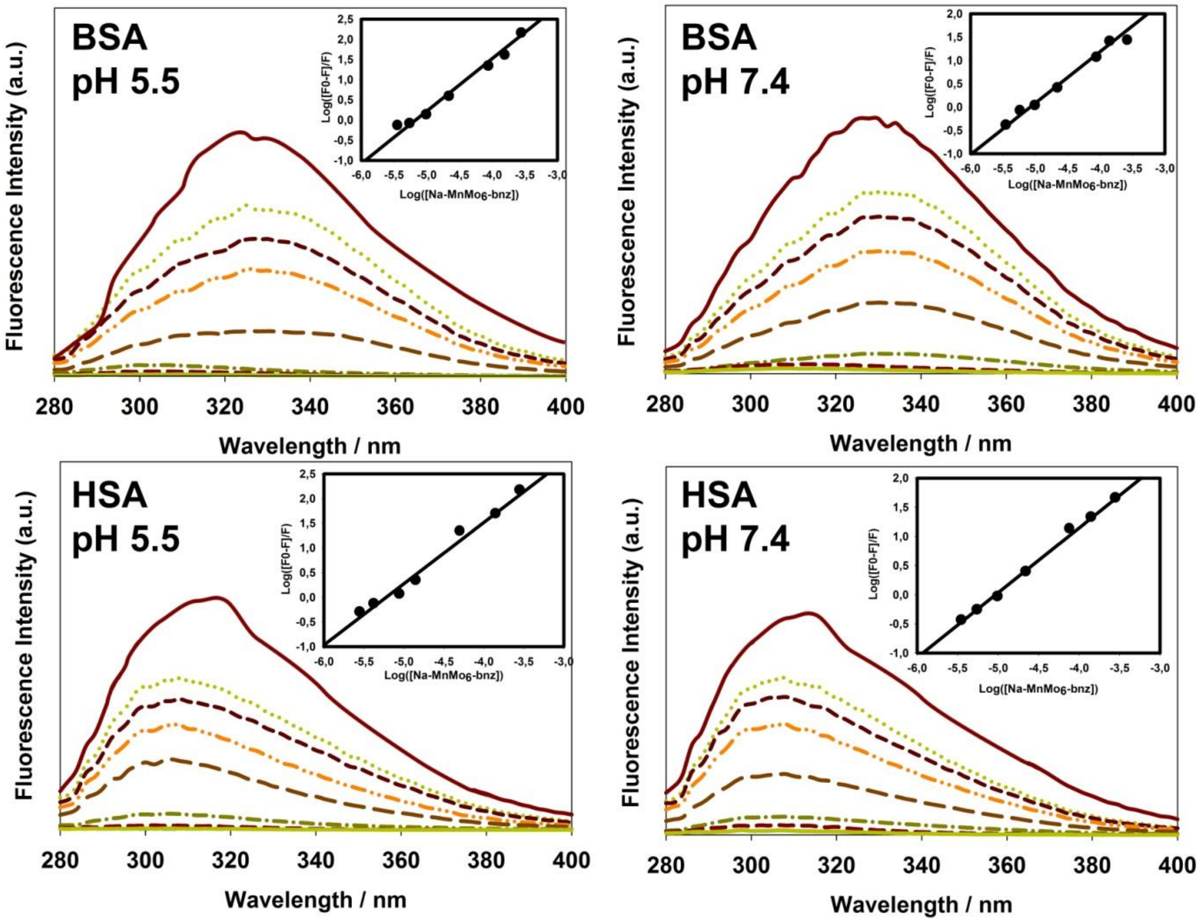

Figure S8. Emission fluorescence spectra of **Na−FeMo$_6$−bnz** with BSA/HSA [BSA]/ [HSA] = 10^{-5} M^{-1}, in 10 mM NaOAc buffer. A: BSA pH 5.5; B: BSA pH 7.4; C: HSA pH 5.5; D: HSA pH 7.4. The top line in each spectrum is collected in the absence of **Na−FeMo$_6$−bnz** followed by stepwise increase (0.006, 0.012, 0.025, 0.05, 0.1, 0.2 and 0.4 fold of **Na−FeMo$_6$−bnz**). In the inset, the plot of the derived Stern–Volmer equation is depicted (with R_2 = 0.99).

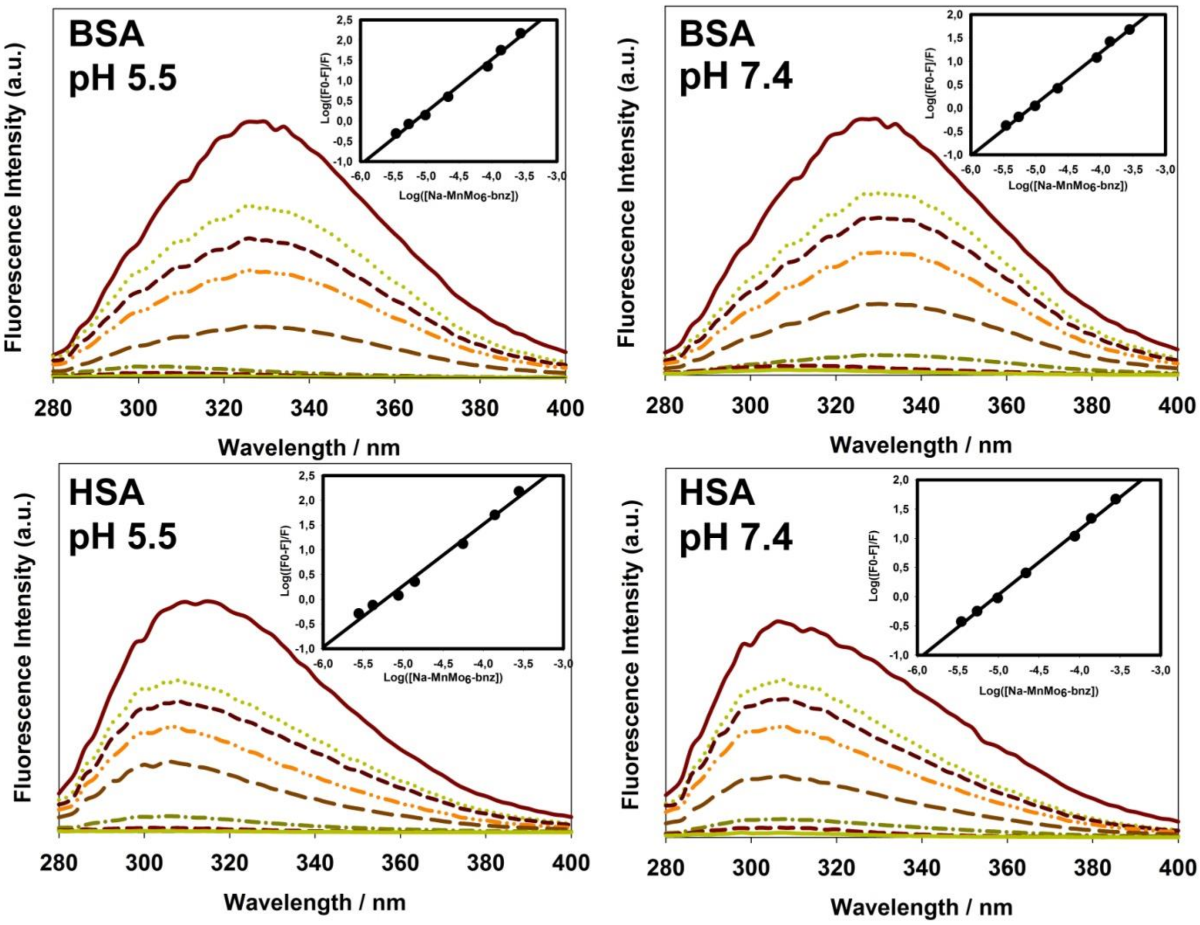

Figure S9. Emission fluorescence spectra of **Na−MnMo$_6$−bnz** with BSA/HSA [BSA]/ [HSA] = 10^{-5} M^{-1}, in 10 mM NaOAc buffer. A: BSA pH 5.5; B: BSA pH 7.4; C: HSA pH 5.5; D: HSA pH 7.4. The top line in each spectrum is collected in the absence of **Na−MnMo$_6$−bnz** followed by stepwise increase (0.006, 0.012, 0.025, 0.05, 0.1, 0.2 and 0.4 fold of **Na−MnMo$_6$−bnz**). In the inset, the plot of the derived Stern–Volmer equation is depicted (with R_2 = 0.99).

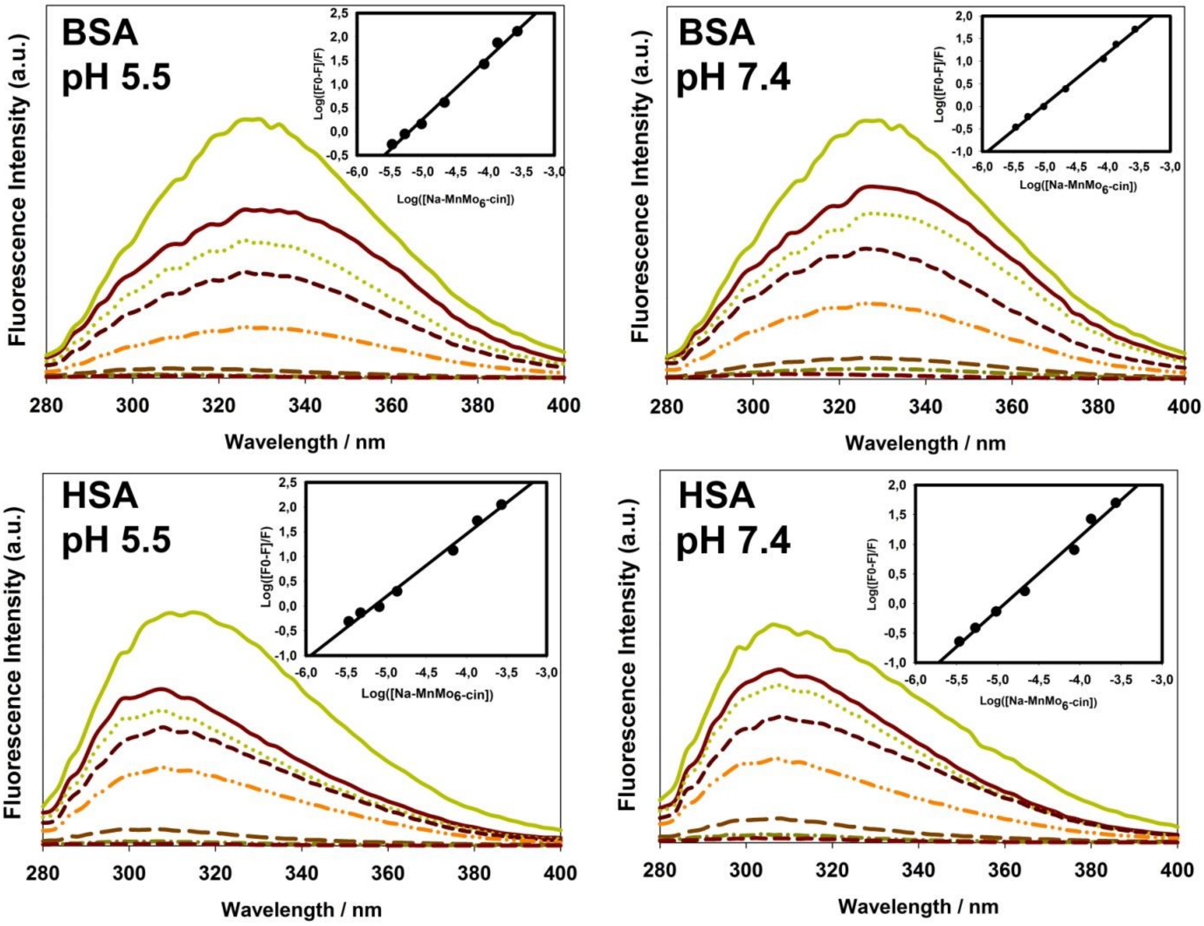

Figure S10. Emission fluorescence spectra of **Na−MnMo$_6$−cin** with BSA/HSA [BSA]/ [HSA] = 10^{-5} M^{-1}, in 10 mM NaOAc buffer. A: BSA pH 5.5; B: BSA pH 7.4; C: HSA pH 5.5; D: HSA pH 7.4. The top line in each spectrum is collected in the absence of **Na−MnMo$_6$−cin** followed by stepwise increase (0.006, 0.012, 0.025, 0.05, 0.1, 0.2 and 0.4 fold of **Na−MnMo$_6$−cin**). In the inset, the plot of the derived Stern–Volmer equation is depicted (with R_2 = 0.99).

7. References

1. U. K. Laemmli, *Nature*, 1970, **227**, 680-685.
2. M. T. Pope, Springer, Heidelberg, 1983;, *Heteropoly and Isopoly Oxometalates*, Springer, 1983.
3. J. Chen, G. Song, Y. He and Q. Yan, *Microchim Acta*, 2007, **159**, 79-85.
4. G. M. Sheldrick, *Acta Crystallogr., Sect. A: Found. Crystallogr.*, 2008, **64**, 112-122.
5. O. V. Dolomanov, L. J. Bourhis, R. J. Gildea, J. A. K. Howard and H. Puschmann, *J. Appl. Crystallogr.*, 2009, **42**, 339-341.
6. A. Spek, *Acta Cryst.*, 2009, **D65**, 148-155.
7. H. S. Rho, H. S. Baek, D. H. Kim and I. S. Chang, *Bull. Korean Chem. Soc.*, 2006, **27**, 584-586.
8. B. Hasenknopf, R. Delmont, P. Herson and P. Gouzerh, *Eur. J. Inorg. Chem.*, 2002, 1081-1087.
9. B. Zhang, L. Yue, Y. Wang, Y. Yang and L. Wu, *Chem. Comm.*, 2014, **50**, 10823-10826.
10. K. Nomiya, T. Takahashi, T. Shirai and M. Miwa, *Polyhedron*, 1987, **6**, 213-218.
